# A28 RESPONSE RATE OF VERY EARLY ONSET INFLAMMATORY BOWEL DISEASE (VEOIBD) PATIENTS TO ANTI-TNF TREATMENT IS HIGHER THAN PREVIOUSLY REPORTED, BUT HIGHER DOSING IS REQUIRED-A LARGE TERTIARY SINGLE CENTER EXPERIENCE

**DOI:** 10.1093/jcag/gwab049.027

**Published:** 2022-02-21

**Authors:** A Eindor, L Meleady, K Jacobson

**Affiliations:** 1 Pediatric Gastroenterology, BC Children’s Hospital, Vancouver, BC, Canada; 2 BC Children’s Hospital, Vancouver, BC, Canada

## Abstract

**Background:**

Very early onset inflammatory bowel disease (VEOIBD) patients are known to have special disease characteristics. Limited data is available regarding biologic use and long-term outcomes in VEOIBD patients.

**Aims:**

We aimed to assess long-term outcomes and time to progression to biologic treatment in VEOIBD patients. Secondary aim was to determine Infliximab prescribing practice during the course of treatment.

**Methods:**

We retrospectively reviewed IBD patients diagnosed under 6 years of age, between January 2005 and December 2019, from the British Columbia (BC) Pediatric IBD database. Demographic data, disease characteristics, symptoms at diagnosis, disease location and severity were documented. Data on biologic treatment at initiation and during follow up including type of biologic, dosing and response were collected. Kaplan Meier curves were used to assess the number of years to progression to biologic treatment and the parameters influencing commencement. Infliximab dosing and intervals were recorded for all patients treated with Infliximab during induction, after one year and at last follow up.

**Results:**

89 patients with VEOIBD were diagnosed during the study period. Median age at diagnosis was 3.8 years (IQR 2.6–5.1), 45.3% had Crohn’s disease (CD) and 62.8% were males. Median duration of follow up was 6.39 years (IQR 3.71–10.55). Biologic treatment was started on 39.5% of patients and 7.1% underwent surgery. In patients with CD, the addition of perianal fistulizing disease or stricturing disease was associated with more rapid progression to biologic treatment (p=0.024, p=0.038, respectively). In patients with UC, disease severity (p=0.017) was associated with early initiation of a biologic. The median Infliximab dose at one year was 10 mg/kg (IQR 7.5–11) with median dose interval of 4 weeks (IQR 4–6). At the time of the last follow up, 18 patients (72%) maintained treated with Infliximab and had a median dose of 9 mg/kg per dose (IQR 8.1–10) and a median dose interval of 4.5 weeks (IQR 4–6). Clinical remission was reported in 61.8% of patients on their first biologic agent at the last follow up.

**Conclusions:**

Factors influencing earlier progression to biologics were disease severity and disease behaviour. The response rate was higher than previously reported and might be due to higher Infliximab dosing with shorter infusion intervals than standard dosing.

**Funding Agencies:**

None

